# Potential Mechanism of Immune Evasion Associated with the Master Regulator ASCL2 in Microsatellite Stability in Colorectal Cancer

**DOI:** 10.1155/2021/5964752

**Published:** 2021-02-10

**Authors:** Qian Yang, Guowei Huang, Liyan Li, Enmin Li, Liyan Xu

**Affiliations:** ^1^The Key Laboratory of Molecular Biology for High Cancer Incidence Coastal Chaoshan Area, Shantou University Medical College, Guangdong, China; ^2^Institute of Oncologic Pathology, Shantou University Medical College, Shantou, China; ^3^Department of Biochemistry and Molecular Biology, Shantou University Medical College, Guangdong, China

## Abstract

Colorectal cancer (CRC) has two major subtypes, microsatellite instability (MSI) and microsatellite stability (MSS) based on the genomic instability. In this study, using computational programs, we identified 9 master transcription factors (TFs) based on epigenomic profiling in MSS CRC samples. Notably, unbiased gene set enrichment analysis (GSEA) showed that several master TFs were strongly associated with immune-related functions in TCGA MSS CRC tissues, such as interferon gamma (IFN-*γ*) and interferon alpha (IFN-*α*) responses. Focusing to the top candidate, ASCL2, we found that CD8^+^ T cell infiltration was low in ASCL2 overexpressed MSS CRC samples. Compared with other gastrointestinal (GI) cancers (gastric cancer, MSI CRC, and esophageal cancer), ASCL2 is specifically upregulated in MSS CRC. Moreover, we identified 28 candidate genes in IFN-*γ* and IFN-*α* response pathways which were negatively correlated with ASCL2. Together, these results link transcriptional dysregulation with the immune evasion in MSS CRC, which may advance the understanding of immune resistance and contribute to developing novel treatments of MSS CRC.

## 1. Introduction

CRC is the third most common cancer and the fourth most common cause of cancer-related death worldwide [1]. Noninvasive biomarkers have been proposed in the diagnosis of CRC [2]. Metastasis of CRC remains the principal cause of mortality [3]. Thus, developing more effective treatments for CRC patients is an urgent and important task. Genomic instability is a key feature underlying CRC, which classifies this cancer into two main groups, microsatellite instability (MSI) and microsatellite stability (MSS) [4]. MSI status is caused by a hypermutable phenotype due to loss of DNA repair mechanisms, which decreased the ability to repair short DNA chains or tandem [5, 6]. In CRC, about 15% of the patients have the MSI phenotype with the mutations of MLH1, MLH3, MSH2, MSH3, MSH6, and PMS2 in DNA mismatch repair pathway [7]. Clinically, MSI cancers have the following characteristics: localization in the proximal colon, hereditary form (younger than 50 years) or sporadic form (elderly people), and synchronous occurrence with additional tumors [8]. Patients with MSI CRC have a better prognosis than those patients with MSS [7, 9, 10]. Recently, immune checkpoint blockade (ICB) therapies have shown encouraging results in patients with MSI CRC; however, the response of MSS patients (accounts for about 85% in CRC) to these immunotherapies remains poor [11]. MSI status only accounts for a small group (~15%) of CRC samples; most of the CRC samples are MSS [7, 9, 10]. Thus, urgent needs exist to understand the resistant mechanism of MSS CRC to ICB therapies.

Type I and II interferons have recently emerged as key regulators of tumor response to immunotherapy [12]. Endogenous IFN-*γ* can promote the immune response to primary tumors, but IFN-*γ*-insensitive samples display increased tumorigenicity and evade tumor surveillance mechanisms [13]. Recent evidence indicates that TFs can directly regulate immune responses and then lead to immunosuppression in tumor [14]. For example, MYC can directly bind to the promoters of CD47 and PDL1 and regulate them to involve the recruitment of macrophages and T cells, and then to modify the tumor microenvironment [15, 16].

A small number of the so-called master TFs are critical for orchestrating the gene expression program by controlling (super) enhancers [17, 18]. In particular, these master TFs can control their own transcription and that of other master TFs through forming core transcription regulatory circuitries. For example, OCT4, SOX2, and NANOG collaborate to form regulatory circuitry consisting of autoregulatory and feedforward loops, which cooccupy many target genes in embryonic stem cells [19]. In esophageal squamous cell carcinomas (ESCC), Jiang et al. [20] have characterized *δ*Np63 and SOX2 as master TFs not only form autoregulatory and feedforward loops but also cooccupy and coregulate many target genes in tumor samples. In esophageal adenocarcinoma (EAC), master TFs, ELF3, KLF5, GATA6, and EHF can promote the expression of each other by interacting with each super enhancer, and also occupied most super enhancers and cooperatively orchestrated the transcriptome [21]. With the development of bioinformatics, master TFs can be identified by an available tool, coltron, which reconstructs the core transcription regulatory circuitry models and then provides the list of master TFs based on (super) enhancers in each sample (https://pypi.org/project/coltron/).

In this study, we aim to identify master TFs based on ChIP-Seq and RNA-Seq data using coltron and explore if these master TFs have association with immune-related functions (e.g., IFN-*γ* and IFN-*α* responses). Then, we will predict the potential mechanism of immune evasion in MSS CRC samples.

## 2. Materials and Methods

### 2.1. Sample Collection and ChIP-Seq Data Analysis

ChIP-Seq data of 35 colorectal primary cell lines were downloaded online from the published study [22], including 25 MSS CRC primary cell lines, 6 MSI CRC primary cell lines, and 4 normal colorectal primary cell lines. For comparing CRC with other GI cancers, we also collected the esophageal and gastric cancers ChIP-Seq data from us and others' studies (18 gastric cancer samples, 11 EAC, and 6 ESCC) [20, 21, 23]. Here, the esophageal cancer samples included 11 EAC tissues and 6 ESCC cell lines we published before [20, 21]. The gastric cancer samples included 18 tumor samples collected from others' studies [23]. Briefly, 50 bp single-end reads of ChIP-Seq data were aligned to human reference genome (HG19) using Bowtie 2 (v2.2.6) (*k* = 2) [24]. Then, we used Picard MarkDuplicates tool to mark the PCR duplicates in each sample (picardtools MarkDuplicates I = input. bam O = output. mkdup.bam M = output. mkdup.txt), and the ENCODE blacklisted regions (https://sites.google.com/site/anshulkundaje/projects/blacklists) were also removed (bedtools intersect -v -a output.mkdup.bam -b blackList.bed > output.mkdup.rmblacklist.bam). Then, model-based analysis for ChIP-Seq (Macs2: v2.2.7.1) was utilized to identify the peaks with the parameters --bdg --SPMR --nomodel --extsize 200 -q 0.01 in each sample [25]. bigWig files were generated by bamCompare in DeepTools (v3.1.3) using parameters --binSize 10 --numberOfProcessors 5 --scaleFactorsMethod None --normalizeUsing CPM --ignoreDuplicates --extendReads 200 from Ramírez et al., 2014 [26]. The bigWig files were then visualized in Integrative Genomics Viewer (IGV) software [27].

### 2.2. Enhancer Annotation and Identification of Core Transcription Regulatory Circuitry

Rank Order of Super Enhancers (ROSE) method [17, 28] was used to identify enhancers which defined as H3K27Ac peaks 2 kb away from any transcription start site (TSS) based on narrowpeaks from Macs2. Following stitching enhancer elements together when clustered within a distance of 12.5 kb, typical enhancer (TE) and super enhancer (SE) were then classified using a cutoff at the inflection point (tangent slope = 1) based on the ranking order.

Core transcription regulatory circuitry is consisted with master TFs and their interconnected autoregulatory loops [19]. The identification and characterization of core transcription regulatory circuitries can contribute to understanding and revealing the crucial biologic phenomenon. In the present study, core transcription regulatory circuitries and master TFs in CRC samples were identified based on an established methodology, coltron, which reconstructs core transcription regulatory circuitry models based on SE-associated master TFs in each sample. This approach can predict core transcription regulatory circuitries and master TFs for diseases (https://pypi.org/project/coltron/). The master TFs of MSS CRC patients were obtained in the output files with in- and out-degree. Finally, we calculated the enrichment score of each master TF based on the published approach [29].

### 2.3. Unbiased Gene Set Enrichment Analysis (GSEA)

RNA-Seq (level 3) data of all cancer types were downloaded from TCGA (exclude the cancer types with the number of samples less than 70). In addition, three CRC datasets were downloaded from Gene Expression Omnibus (https://www.ncbi.nlm.nih.gov/geo/): GSE13294, GSE13067, and GSE35896 [30, 31]. Here, we first ranked tumor samples in each cancer type based on the expression of each master TF and then classified the samples into two groups (TF high (top 30% samples) and TF low (bottom 30% samples)). We treated MSS and MSI CRC samples as two datasets and did the classification analysis separately. Secondly, differentially expressed genes were determined using the limma method [32]. Finally, 50 hallmark gene sets from Molecular Signatures Database were used and we performed GSEAPreranked to identify the significant hallmark pathways related to the master TFs [33].

### 2.4. Expression of Master TFs in CCLE

We downloaded RNA expression of all cancer cell lines from CCLE database [34]. Then, we extracted the expression of the master TFs and showed the expression among all cancer types. Here, we treated MSS and MSI CRC cell lines as two types of cancer cell and sorted all types of human cancer cell lines by the average expression of each master TF.

### 2.5. T Cell Infiltration-Related Gene Signatures and TIL Scores

T cell infiltration-related gene signatures were identified by some established approaches. The first gene signature included twelve canonical T cell-associated genes (*CCL2*, *CCL3*, *CCL4*, *CXCL9*, *CXCL10*, *CD8A*, *HLA-DOB*, *HLA-DMB*, *HLA-DOA*, *GZMK*, *ICOS*, and *IRF1*), which identified by analyzing the genetic drivers of immune evasion based on about 1000 CRC samples [35]. The average log expression of twelve canonical T cell-associated genes is defined as “T cell average” for each sample with expression data. The second gene signature was extracted by Tumor IMmune Estimation Resource (TIMER) database, which systematically evaluates the abundance of six immune cell types: B cell, CD4 T cell, CD8 T cell, neutrophil, macrophage, and dendritic cell in the tumor microenvironment based on a novel statistical method and about 4000 microarray RNA expression data [36]. Here, we calculated the CD8^+^ T cell scores based on TCGA COAD RNA-Seq using TIMER. The immune cytolytic activity (CYT) score is calculated based on the average expression of GZMA and PRF1 [37] (not present in the twelve canonical T cell-associated genes), which is the third gene signature we used in this study. Tumor-infiltrating lymphocyte (TIL) score is a pathology-based measure of T cell infiltration based on 429 histology pathology slides of TCGA COAD samples. We obtained the TIL scores of TCGA COAD samples from the published paper [38]. All abbreviations and the corresponding full names are listed in Supplementary Table 2.

### 2.6. Statistical Analysis

Pearson correlation was calculated using cor.test() function in R software (3.6.3).*t*‐test() was used to calculate the significance in different groups in the boxplots.

ChIP-Seq data were downloaded on March 1, 2019. The GEO datasets were downloaded on July 15, 2019. The TCGA and CCLE RNA-Seq data in this study were released on March 26, 2019 (GDC V16.0) and January 2, 2019, respectively.

## 3. Results

### 3.1. Master TFs in MSS CRC Based on ChIP-Seq and RNA-Seq Data

**Figure 1:**
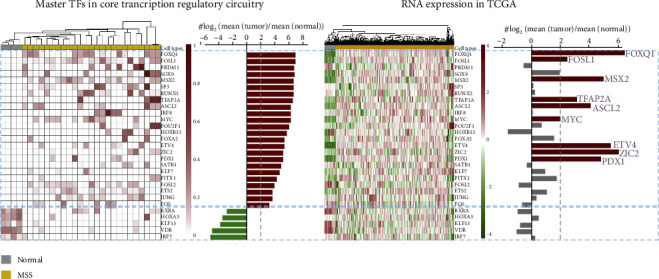
Master TFs in MSS CRC. (a) Master TFs in core transcription regulatory circuitries in MSS CRC primary cells. (b) RNA expression of these master TFs in TCGA COAD samples.

To identify candidate master TFs from MSS CRC samples, the coltron method was first performed using the published H3k27ac ChIP-Seq data based on enhancers from ROSE. A total of 55 TFs were identified in CRC core transcription regulatory circuitries. Based on the enrichment score, top 24 activated and 5 deactivated TFs are showed in Figure 1(a) (log_2_ (fold change (enrichment score)) > 2). For further filtration, average enrichment score of upregulated TFs and TCGA COAD RNA-Seq data was used. Finally, 9 upregulated master TFs (FOXQ1, ZIC2, ETV4, MSX2, PDX1, ASCL2, TFAP2A, FOSL1, and MYC) were identified in MSS CRC samples (Figure 1) (cutoffs : log_2_ (fold change (expression)) > 2 and max (enrichment score) > 0.2). Some of the candidate master TFs were known CRC-promoting factors, such as MYC, PDX1, FOXQ1, and ETV4 [39–41], validating the approach of our method. Importantly, several of these candidates have not been investigated in CRC.

### 3.2. ASCL2 and ETV4 Negatively Correlated with IFN Signal in MSS CRC

**Figure 2:**
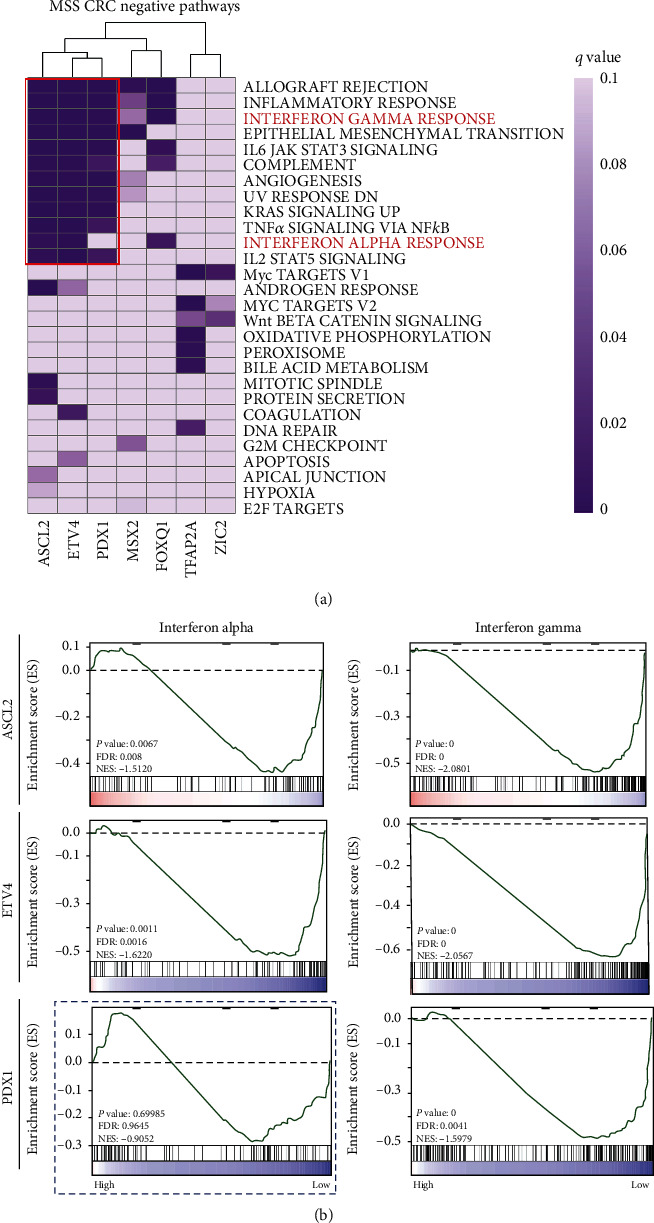
Negative regulated pathways by master TFs. (a) Negative regulated pathways by master TFs in TCGA COAD tissues (MSS). (b) GSEA curves of IFN-*γ* and IFN-*α* response pathways in ASCL2, ETV4, and PDX1 classifications, respectively.

To explore downstream signaling pathways affected by these master TFs predicted above, we performed unbiased GSEA using MSS CRC patient samples in TCGA. We first stratified MSS COAD primary samples into either the TF-high (top 30% samples) or TF-low (bottom 30% samples) groups, based on the expression of each candidate master TF. Next, differentially expressed genes (log_2_ (fold change)) between these two groups were used to perform GSEAPreranked analysis. Notably, the top-ranked negative pathways were all immune response related, such as allograft rejection, inflammatory response, and IFN-*γ* response (Figure 2(a)), which were strongly enriched in the results of ASCL2, ETV4, and PDX1 (Figure 2(b)). No significant negative pathway was obtained based on the classifications of MYC and FOSL1. IFN-*γ* (belongs to interferon type II) is an important activator of macrophages and inducer of class II major histocompatibility complex (MHC) molecule expression [42]. IFN-*α* is mainly involved in innate immunity against viral infection [42]. Recent immunotherapy research shows that IFNs are produced by various cell types in the tumor microenvironment; then, they can affect tumor cells directly or affect via modulation of the immune response indirectly [43]. Therefore, the activation of IFN can benefit to the cancer immunotherapy. We found that ASCL2 and ETV4 showed low normalized enrichment score (NES) and significant *P* values in IFN-*γ* and IFN-*α* response pathways, respectively (Figure 2(b)), while PDX1 had no significant enrichment of IFN-*γ* response pathway (Figure 2(b)). These results indicated that the overexpression of ASCL2 and ETV4 might inhibit the IFN signal in MSS CRC and block the immune response in immunotherapy. Focusing on ASCL2 and ETV4, three independent datasets of MSS CRC samples further confirmed these strong enrichments (Supplementary Figure 1).

### 3.3. Expression of ASCL2 and ETV4 Is Inversely Correlated with T Cell Infiltration in TCGA MSS CRC Cohort

**Figure 3:**
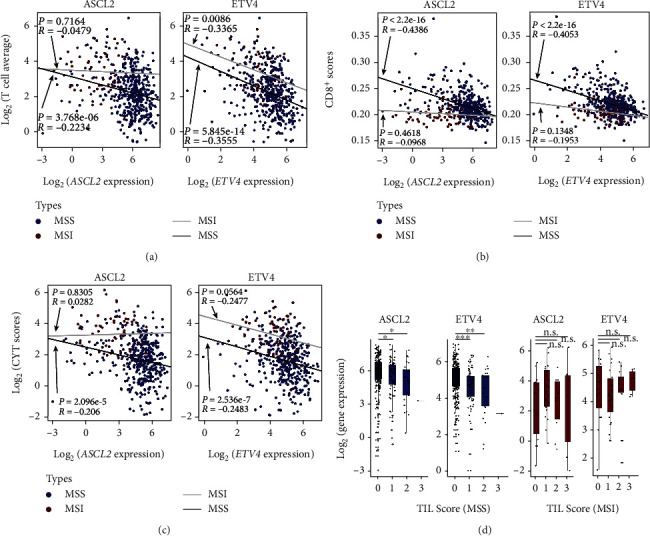
Correlation analysis of ASCL2 and ETV4 with T cell infiltration in TCGA COAD cohort. (a) Correlation analysis of ASCL2 (ETV4) and twelve canonical T cell-associated genes. (b) Correlation analysis of ASCL2 (ETV4) and CD8^+^ scores. (c) Correlation analysis of ASCL2 (ETV4) and CYT scores. (d) Box plot of TIL scores and ASCL2 (ETV4) in MSI and MSS samples, respectively. *x*-axis is the TIL score and *y*-axis is the expression of certain TF. ^∗^*P* < 0.05; ^∗∗^*P* < 0.01. ns: no significance.

The above results suggest that the upregulation of ASCL2 and ETV4 is associated with the inhibition of IFN pathway in MSS patients. We next investigated the correlation between the expression of ASCL2 and ETV4 and the T cell infiltration-associated gene signatures. Three independent gene signatures of T cell infiltration, including T cell average, CYT scores, and CD8^+^ scores, were calculated for the TCGA COAD samples (see Materials and Methods). In T cell average gene signature, the expression of ASCL2 and ETV4 was highly negatively correlated with twelve canonical T cell infiltration-associated genes (R_ASCL2 = −0.22 and R_ETV4 = −0.35) (Figure 3(a)). Since CD8^+^ T cells play a central role in immune response, CD8^+^ T cell scores can reflect the T cell activity in tumor samples. Based on the statistical method in TIMER database, we found the expression of ASCL2 and ETV4 had prominent anticorrelation with CD8^+^ scores (R_ASCL2 = −0.44 and R_ETV4 = −0.41) (Figure 3(b)), which indicated that increased ASCL2 and ETV4 might be associated with the decreased abundance of CD8^+^ T cells in the MSS tumor microenvironment. Another measurement of immune cytolytic activity and evasion is CYT score, which focuses on the transcript levels of two key cytolytic effectors, GZMA and PRF1, using RNA-Seq data from TCGA solid tumor biopsies [37]. These two genes are dramatically upregulated upon CD8^+^ T cell activation and during productive clinical responses to anti-PD-L1 immunotherapies [44]. Consistent with the above results, ASCL2 and ETV4 are negatively associated with CYT scores in MSS CRC ((R_ASCL2 = −0.21 and R_ETV4 = −0.25)) (Figure 3(c)). In contrast, there is no significant negative correlation of these two master TFs with T cell infiltration in MSI samples (Figures 3(a), 3(b), and 3(c)).

In addition to the above three gene signatures based on mRNA expression, we also collected TIL scores, a pathology-based measure of T cell infiltration, based on 429 histology slides from TCGA COAD samples [38]. Consistently, samples with higher TIL scores expressed lower levels of ASCL2 and ETV4 in MSS CRC samples (Figure 3(d)). In contrast, no such anticorrelation was observed in MSI CRC samples (Figure 3(d)). Together, these results showed that the expression of ASCL2 and ETV4 had negative correlation with T cell infiltration in the tumor microenvironment, indicating that overexpression of ASCL2 and ETV4 might inhibit the recruitment and activation of CD8^+^ T cells in MSS CRC patients.

### 3.4. Overexpressed ASCL2 Might Induce Immune Evasion in MSS CRC

**Figure 4:**
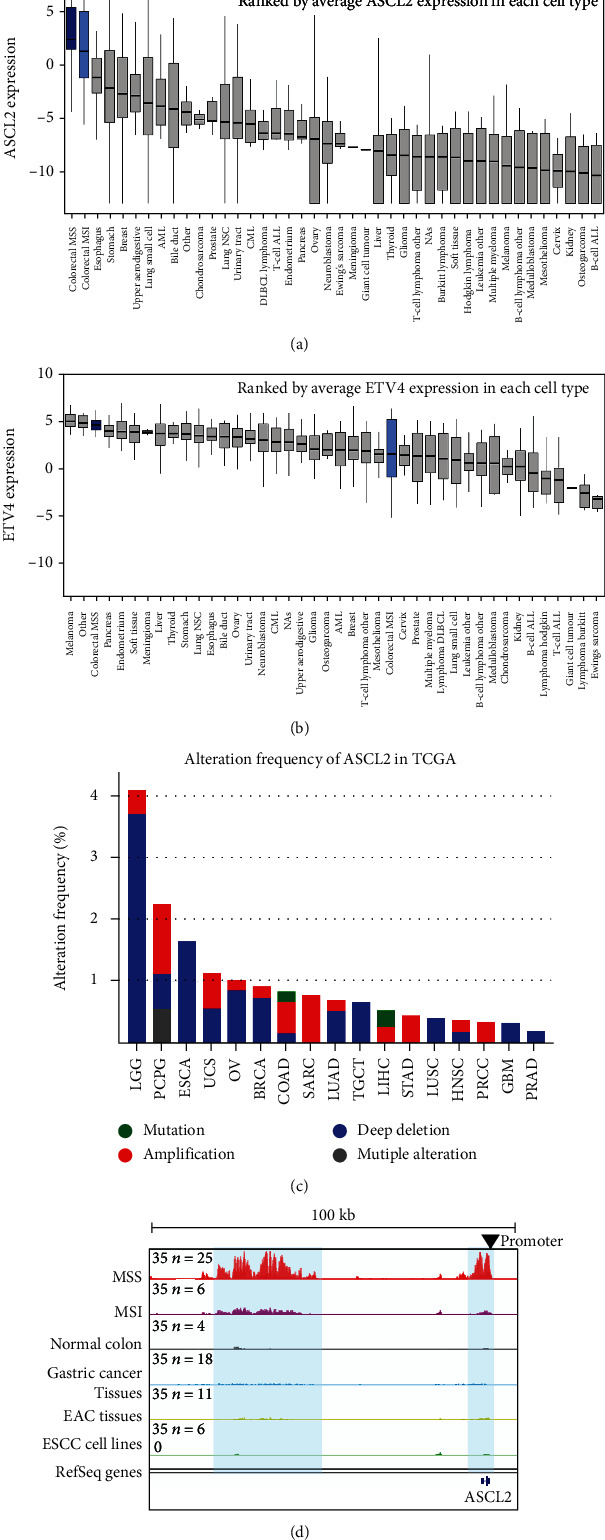
Expression and alteration of ASCL2. (a) Expression of ASCL2 in all CCLE human cancer cell lines. (b) Expression of ETV4 in all CCLE human cancer cell lines. (c) Alteration of ASCL2 in TCGA cancer types. (d) IGV tracks of common GI cancers in ASCL2 locus (H3K27Ac ChIP-Seq data).

Considering that ASCL2 and ETV4 are upregulated in MSS CRC tissues in TCGA, we next investigated the expression of ASCL2 and ETV4 in MSS CRC cell lines and compared their expression with other cancer cell lines. Notably, MSS CRC cell lines had the highest expression of ASCL2 among all human cancer cell lines (Figure 4(a)). In contrast, ETV4 showed a ubiquitous expression pattern (Figure 4(b)). In addition, rare genomic amplification was observed at ASCL2 locus (Figure 4(c)). Amplification only accounts for 0.51% (3 cases) in TCGA COAD samples. Mutation and deep deletion account for 0.17% (1 case) and 0.17% (1 case) in TCGA COAD samples, respectively [45]. ASCL2 has no alternation in TCGA COAD samples (Figure 4(c)). To explore whether ASCL2 is activated epigenomically, we compared MSS CRC with MSI CRC, gastric cancer, EAC, and ESCC. Here, the MSI and normal colorectal ChIP-Seq data came from the same study with MSS ChIP-Seq data (Figure 4(d)). Gastric cancer ChIP-Seq data was downloaded from the published paper [23]. Both of EAC tissues and ESCC cell lines ChIP-Seq data are the published data from our group [21, 46]. Notably, H3K27Ac signals of ASCL2 were uniquely high in MSS CRC samples (Figure 4(d)). In fact, this was annotated as super enhancer, supporting that overexpression of ASCL2 in MSS CRC was due to epigenomic activation.

**Figure 5:**
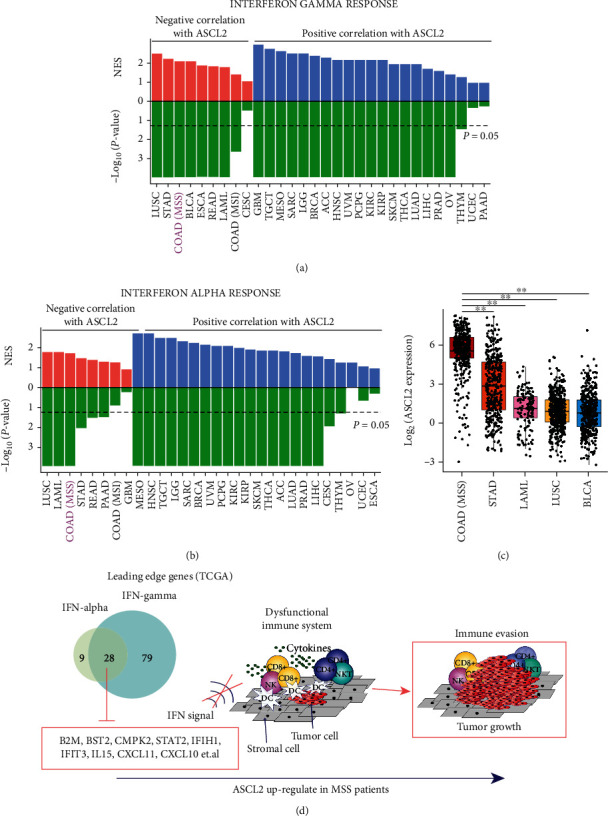
Potential mechanism of immune evasion in MSS CRC. (a) NES and *P* values of IFN-*γ* response in 30 cancer types in TCGA. (b) NES and *P* values of IFN-*α* response in 30 cancer types in TCGA. (c) Expression of ASCL2 in TCGA COAD (MSS), STAD, LAML, LUSC, and BLCA samples. (d) Potential mechanism of immune evasion in MSS CRC. ^∗∗^*P* < 0.01.

Moreover, we classified all cancer samples (in each cancer type) in TCGA based on the RNA expression of ASCL2. Among the 30 types of cancers, MSS CRC ranks the 3^rd^ negative NES and lowest *P* value in IFN-*γ* response (Figure 5(a)). In IFN-*α* response, MSS CRC is the 4^th^ one, followed by gastric cancer (Figure 5(b)). Although the expression of ASCL2 is negatively correlated with IFN-*γ* and IFN-*α* response pathways in several cancer types, COAD MSS samples have the highest expression of ASCL2 among these cancer types (Figure 5(c)). Unbiased GSEA analysis results further implied that the inhibition of IFN-*γ* and IFN-*α* responses might explain the mechanism of low infiltration and immune evasion in the MSS status tumor microenvironment (Figure 5(d)). To understand how ASCL2 inhibits the IFN-*γ*/*α* responses, leading edge genes were extracted in the IFN-*γ* and IFN-*α* responses from unbiased GSEA results. Twenty-eight overlap leading edge genes (based on TCGA COAD RNA-Seq data) were identified in these two pathways, which might be suppressed by ASCL2 in MSS CRC (Figure 5(d)). IFN-*α*, as a part of type I IFNs, has important fundamental and clinical implications for antitumor immunity [47]. The activation of IFN-*γ* can enhance their recognition by CD8 T cells as well as by CD4 T cells, and also unveil a key role in the promotion of tumor immunogenicity [48]. These 28 predicted genes in IFN signal encompass important inflammatory signaling molecules, transcriptional activators, and cell cycle and apoptosis regulators (Supplementary Table 1). For example, interleukin-15 (IL-15) is a pleiotropic cytokine with extensive biological functions in different cell types, which plays a major role in the development of inflammatory response and modulating immune system [49]. The secretion of IL15 can promote the infiltration of immune cells with antitumor activity in CRC [50]. Another predicted gene, STAT2, is an important TF belongs to STAT family, which can be activated by multiple growth factors and cytokines [51]. Defects in the expression or nuclear localization of STAT2 can lessen the efficacy of IFN-related immunotherapies [51].

## 4. Discussion

In CRC, genomic instability determines the response of immunotherapy [6]. Patients with mismatch repair-deficient (dMMR) or MSI status (accounts for ~15% in CRC) have benefit immune checkpoint therapy response. However, pMMR or MSS CRC patients (account for ~85% in CRC) have low tumor mutation burden and immune cell infiltration, which have been posited as mechanisms of immune resistance [52, 53]. Moreover, master TFs can control the expression of not only themselves but also their target genes by forming core transcription regulatory circuitries in cancers [17, 18, 20]. Therefore, the present work is aimed at identifying the master TFs and predicting the potential mechanism of tumor immune evasion through bioinformatics analysis in MSS CRC samples (a large proportion of CRC) based on ChIP-Seq and RNA-Seq data. These analysis results may provide the effective treatment strategies of this deadly cancer type. Based on this goal, we first collected the ChIP-Seq data of 35 CRC primary cells from the published paper [22] and RNA expression data (level 3) of COAD from TCGA database (https://www.cancer.gov/tcga). Then, master TFs in MSS patients were identified based on the ChIP-Seq data using established methods, ROSE and coltron. These master TFs as particularly activated genes exist in most of core transcription regulatory circuitries in MSS CRC patients (Figure 1(a)). We further filtrated these master TFs by RNA expression data which were upregulated significantly in the MSS groups compared with normal colorectal samples (Figure 1(b)). The identification and characterization of master TFs in core transcription regulatory circuitries can contribute to revealing the important pathophysiological mechanism of MSS CRC.

Next, to explore the downstream functions of master TFs, we performed unbiased GSEA analysis based on RNA expression data in TCGA. The results show that highly expressed master TFs were negatively associated with immune response-related functions in MSS samples. Specifically, IFN-*γ* and IFN-*α* responses were existed simultaneously in two master TFs (ASCL2 and ETV4) classification results with low NES scores and significant *P* values (Figures 2(a) and 2(b)). Unbiased GSEA analysis in three independent MSS CRC gene expression datasets further confirmed the strong enrichment results (Supplementary Figure 1). Recently, IFNs, especially IFN-*γ*, have been reported to have important roles in tumor immunotherapy [54]. IFN-*γ* is mainly produced by NK cells and T cells in response to multiple inflammatory or immune stimuli. TILs are the main source of IFN-*γ*, which are particularly important in tumor immunosurveillance [55]. ICB therapy can upregulate the IFN-*γ* response and in turn scavenge tumor cells [56], while IFN-*γ*-insensitive patients display increased immune evasion and resistance to immunotherapy [13]. In addition, IFN-*α* has also emerged as central coordinators of tumor immune system [55]. High-dose IFN-*α* immunotherapy can significantly increase immune response and overall survival time compared with untreated patients [57]. The unbiased GSEA results indicated that overexpression of ASCL2 and ETV4 might reduce the immune response and led to the immune evasion through blocking the IFN-*γ* and IFN-*α* responses in MSS CRC.

To further confirm ASCL2 and ETV4 are associated with the effectiveness of immunotherapy, three T cell infiltration-related gene signatures and TIL scores were used. Correlation analysis suggests that ASCL2 and ETV4 might reduce immune infiltration (Figures 3(a), 3(b), and 3(c)), which might explain the mechanism of immune evasion and resistance to immunotherapy in MSS CRC. In addition, overexpressed ASCL2 and ETV4 MSS samples with low TIL scores further confirmed these two master TFs played important roles in IFN-*γ* and antitumor immune response (Figure 3(d)). However, there is no antiassociation between ASCL2 (also ETV4) and TIL scores in MSI CRC samples. Notably, among all cell lines in CCLE, MSS CRC cell lines have the highest ASCL2 expression (Figure 4(a)), which also reminds that ASCL2 might play important roles in this cancer type. In contrast, ETV4 showed a ubiquitous expression pattern among all human cancer cell lines (Figure 4(b)). Here, we infer that not only in CRC with MSS status but also in other cancer types, such as melanoma and other GI cancers, ETV4 may act as key functions (Figure 4(b) and Supplementary Figure 2). Moreover, H3K27Ac signal on ASCL2 also suggests the importance of ASCL2 in MSS CRC compared with other common GI cancers (Figure 4(d)). H3K27Ac signal on ETV4 indicates that ETV4 might be a common upregulated TF in other GI cancers (Supplementary Figure 2). Finally, twenty-eight ASCL2 negatively associated genes were predicted in IFN-*γ* and IFN-*α* response pathways, which might help us to comprehensively understand the deficient IFN response in MSS samples and contribute to better designing the combination of anticancer drugs and immunotherapies. In the meanwhile, this study identified ASCL2 as a master regulator in MSS CRC using bioinformatic approach based on the published datasets (GI cancer ChIP-Seq data and TCGA pan-cancer RNA-Seq data). More functional experiments in vivo and in vitro need to be done in the future.

Clinically, the response of MSS CRC patients to immune checkpoint blockade (ICB) therapies remains poor [11]. The findings of this study indicated that the expression of ASCL2 might influence the immune evasion in MSS patients, which may advance the understanding of poor ICB therapies to MSS CRC patients clinically. Targeting ASCL2 and enhancing the expression of the 28 IFN-related genes might contribute to improving the effect of immunotherapy in MSS CRC patients.

## 5. Conclusions

In conclusion, this study identifies ASCL2 as a specific master TF in CRC with MSS status, whose presence is significantly negatively correlated with IFN-*γ* and IFN-*α* responses in the tumor microenvironment. Unbiased GSEA analysis, independent datasets, T cell infiltration-related gene signatures, and TIL scores further reveal that ASCL2 could be a “bystander” gene associated with impaired IFN-*γ* response, IFN-*α* response, and T cell infiltration. Finally, we predicted 28 leading edge genes in the IFN pathway associated with ASCL2 expression, which may contribute to revealing the potential mechanism of immune resistance and the management of novel clinical immunotherapy approaches in MSS CRC patients. Further studies are required to understand how ASCL2 regulates IFN-*γ* and IFN-*α* responses within the tumor microenvironment, which might prevent the immune evasion and improve the effect of immunotherapies in MSS CRC patients.

## Data Availability

All data used to support the findings of the present study are described in Materials and Methods.

## Supplementary Materials

Supplementary Materials Supplementary Figure 1: unbiased GSEA results of ASCL2 and ETV4 based on three independent datasets of MSS CRC. Supplementary Figure 2: IGV of ETV4 locus in common GI cancers. Supplementary Table 1: leading edge genes in IFN-*γ* and IFN-*α* response pathways based on ASCL2 and ETV4 classification. Supplementary Table 2: abbreviation list ranked by the present order.
