# Supplementary material for: Potential Mechanism of Immune Evasion Associated with the Master Regulator ASCL2 in Microsatellite Stability in Colorectal Cancer
Source: J Immunol Res. 2021 Feb 10;2021:5964752. doi: 10.1155/2021/5964752 (PMC7892217; doi:10.1155/2021/5964752)
Supplement: Supplementary Materials — Supplementary Figure 1: unbiased GSEA results of ASCL2 and ETV4 based on three independent datasets of MSS CRC. Supplementary Figure 2: IGV of ETV4 locus in common GI cancers. Supplementary Table 1: leading edge genes in IFN-γ and IFN-α response pathways based on ASCL2 and ETV4 classification. Supplementary Table 2: abbreviation list ranked by the present order. [file 5964752.f1.zip › Supplementary table 1.docx]

**Supplementary table 1.** Leading edge genes in IFN-γ and IFN-α response pathways based on ASCL2 and ETV4 classification.

| ASCL2-IFN-γ | ASCL2-IFN-α | ETV4-IFN-γ | ETV4-IFN-α |
| --- | --- | --- | --- |
| IRF2 | OASL | PELI1 | OASL |
| PLSCR1 | ADAR | CASP7 | CMPK2 |
| ISG20 | HERC6 | LATS2 | SP110 |
| NLRC5 | MX1 | NFKBIA | IFIH1 |
| TNFAIP2 | NCOA7 | MX2 | ISG20 |
| TNFAIP3 | RTP4 | OASL | B2M |
| SAMHD1 | B2M | CMPK2 | UBE2L6 |
| SECTM1 | CSF1 | SAMHD1 | IFI30 |
| MX2 | WARS | NLRC5 | MX1 |
| SELP | BST2 | TNFAIP3 | CSF1 |
| HLA-DMA | CMPK2 | SP110 | STAT2 |
| HLA-DRB1 | EIF2AK2 | IFIH1 | PARP9 |
| IL18BP | LPAR6 | ARID5B | RTP4 |
| OASL | PARP9 | MT2A | WARS |
| ADAR | USP18 | ISG20 | TXNIP |
| ST3GAL5 | LAMP3 | B2M | IFIT2 |
| HERC6 | STAT2 | IL18BP | IL15 |
| PSMA2 | IFIH1 | GCH1 | BST2 |
| MX1 | IFIT3 | UBE2L6 | GBP2 |
| RTP4 | IL15 | IFI30 | IFIT3 |
| NAMPT | C1S | XCL1 | PARP14 |
| B2M | IFIT2 | SOCS3 | USP18 |
| BPGM | PARP14 | MX1 | CD74 |
| TNFSF10 | SP110 | STAT2 | DDX60 |
| ITGB7 | TXNIP | IL6 | RSAD2 |
| RAPGEF6 | GBP2 | RTP4 | C1S |
| OAS3 | SELL | ST3GAL5 | IFI44 |
| WARS | RSAD2 | WARS | SAMD9 |
| KLRK1 | IFI44 | DDX58 | TENT5A |
| C1R | CXCL10 | ICAM1 | IFI44L |
| PTPN2 | CXCL11 | SOD2 | CXCL11 |
| CASP7 | TENT5A | TXNIP | SELL |
| BST2 | DDX60 | IFIT2 | SAMD9L |
| CMPK2 | IFI44L | IL15 | GBP4 |
| CD38 | GBP4 | HLA-DMA | CXCL10 |
| IFIT1 | SAMD9 | SECTM1 |  |
| EIF2AK2 | SAMD9L | APOL6 |  |
| ICAM1 |  | BST2 |  |
| SPPL2A |  | IFIT1 |  |
| ARID5B |  | C1R |  |
| IL2RB |  | TNFAIP2 |  |
| CCL2 |  | STAT1 |  |
| PELI1 |  | IFIT3 |  |
| IRF4 |  | PARP14 |  |
| SOCS3 |  | SELP |  |
| USP18 |  | P2RY14 |  |
| GCH1 |  | USP18 |  |
| SOD2 |  | SSPN |  |
| STAT2 |  | CCL7 |  |
| IFIH1 |  | EIF4E3 |  |
| IFIT3 |  | CCL2 |  |
| IL6 |  | XAF1 |  |
| GZMA |  | CD74 |  |
| IL15 |  | SERPING1 |  |
| LATS2 |  | PLA2G4A |  |
| FPR1 |  | DDX60 |  |
| STAT1 |  | HIF1A |  |
| C1S |  | HLA-DRB1 |  |
| SERPING1 |  | IL2RB |  |
| BANK1 |  | FCGR1A |  |
| IFIT2 |  | RSAD2 |  |
| CCL7 |  | ITGB7 |  |
| DDX58 |  | C1S |  |
| CIITA |  | IFI44 |  |
| P2RY14 |  | VCAM1 |  |
| IDO1 |  | STAT4 |  |
| PARP14 |  | JAK2 |  |
| FCGR1A |  | GZMA |  |
| SP110 |  | CCL5 |  |
| TXNIP |  | ST8SIA4 |  |
| APOL6 |  | FAS |  |
| SLAMF7 |  | PTGS2 |  |
| CMKLR1 |  | CMKLR1 |  |
| CCL5 |  | CD38 |  |
| STAT4 |  | IFI44L |  |
| RSAD2 |  | CD274 |  |
| EIF4E3 |  | CXCL11 |  |
| IFI44 |  | TNFAIP6 |  |
| IL10RA |  | CFH |  |
| VCAM1 |  | IRF4 |  |
| CD274 |  | PDE4B |  |
| GPR18 |  | CIITA |  |
| CXCL10 |  | FPR1 |  |
| CXCL11 |  | IL10RA |  |
| ST8SIA4 |  | LCP2 |  |
| XAF1 |  | GPR18 |  |
| CSF2RB |  | OAS2 |  |
| CFH |  | CD86 |  |
| HLA-DQA1 |  | CSF2RB |  |
| FGL2 |  | SAMD9L |  |
| LCP2 |  | GBP4 |  |
| CD86 |  | IDO1 |  |
| CD69 |  | CD69 |  |
| SSPN |  | FGL2 |  |
| FAS |  | SLAMF7 |  |
| DDX60 |  | HLA-DQA1 |  |
| IFI44L |  | CXCL10 |  |
| TNFAIP6 |  | CXCL9 |  |
| JAK2 |  |  |  |
| HIF1A |  |  |  |
| GBP4 |  |  |  |
| PLA2G4A |  |  |  |
| PDE4B |  |  |  |
| OAS2 |  |  |  |
| CXCL9 |  |  |  |
| PTGS2 |  |  |  |
| SAMD9L |  |  |  |
