# Supplementary material for: Potential Mechanism of Immune Evasion Associated with the Master Regulator ASCL2 in Microsatellite Stability in Colorectal Cancer
Source: J Immunol Res. 2021 Feb 10;2021:5964752. doi: 10.1155/2021/5964752 (PMC7892217; doi:10.1155/2021/5964752)
Supplement: Supplementary Materials — Supplementary Figure 1: unbiased GSEA results of ASCL2 and ETV4 based on three independent datasets of MSS CRC. Supplementary Figure 2: IGV of ETV4 locus in common GI cancers. Supplementary Table 1: leading edge genes in IFN-γ and IFN-α response pathways based on ASCL2 and ETV4 classification. Supplementary Table 2: abbreviation list ranked by the present order. [file 5964752.f1.zip › Supplementary table 2.docx]

**Supplementary table 2:** Abbreviations list ranked by order of appearance.

| Abbreviations | Full Names |
| --- | --- |
| ASCL2 | Achaete-Scute Family BHLH Transcription Factor 2 |
| MSS | Microsatellite stable |
| CRC | Colorectal cancer |
| MSI | Microsatellite instability |
| TFs | Transcription factors |
| TCGA | The Cancer Genome Atlas |
| GSEA | Gene Set Enrichment Analysis |
| IFN-γ | Interferon gamma |
| IFN-α | Interferon alpha |
| GI | Gastrointestinal |
| pMMR | Mismatch-repair-proficient |
| ICB | Immune checkpoint blockade |
| MYC | Myelocytomatosis |
| CD47 | Cluster of Differentiation 47 |
| PDL1 | Programmed death-ligand 1 |
| OCT4 | Octamer-binding transcription factor 4 |
| SOX2 | Sex determining region Y-box 2 |
| NANOG | Nanog homeobox |
| ESCC | Esophageal squamous cell carcinomas |
| δNp63 | Tumor protein p63 |
| EAC | Esophageal adenocarcinoma |
| ELF3 | E74 like ETS transcription factor 3 |
| KLF5 | Kruppel like factor 5 |
| GATA6 | GATA binding protein 6 |
| EHF | ETS homologous factor |
| ENCODE | Encyclopedia of DNA Elements |
| Macs2 | Model-based Analysis of ChIP-Seq version 2 |
| IGV | Integrative Genomics Viewer |
| ROSE | Rank Order of Super Enhancers |
| TSS | Transcription start site |
| TE | Typical-enhancer |
| SE | Super-enhancer |
| CCLE | Cancer Cell Line Encyclopedia |
| TIL | Tumor-infiltrating lymphocyte |
| CCL2 | C-C motif chemokine ligand 2 |
| CCL3 | C-C motif chemokine ligand 3 |
| CCL4 | C-C motif chemokine ligand 4 |
| CXCL9 | C-X-C motif chemokine ligand 9 |
| CXCL10 | C-X-C motif chemokine ligand 10 |
| CD8A | CD8a molecule |
| HLA-DOB | Major histocompatibility complex, class II, DO beta |
| HLA-DMB | Major histocompatibility complex, class II, DM beta |
| HLA-DOA | Major histocompatibility complex, class II, DO alpha |
| GZMK | Granzyme K |
| ICOS | Inducible T cell costimulator |
| IRF1 | Interferon regulatory factor 1 |
| TIMER | Tumor Immune Estimation Resource |
| COAD | Colon Adenocarcinoma |
| CYT | Immune cytolytic activity |
| GZMA | Granzyme A |
| PRF1 | Perforin |
| FOXQ1 | Forkhead box Q1 |
| ZIC2 | Zic family member 2 |
| ETV4 | ETS variant transcription factor 4 |
| MSX2 | Msh homeobox 2 |
| PDX1 | Pancreatic and duodenal homeobox 1 |
| TFAP2A | Transcription factor AP-2 alpha |
| FOSL1 | FOS like 1, AP-1 transcription factor subunit |
| MHC | Major histocompatibility complex |
| LI15 | Interleukin-15 |
| STAT2 | Signal transducer and activator of transcription 2 |
| dMMR | Mismatch-repair-deficient |
