# Supplementary figures and images for: Potential Mechanism of Immune Evasion Associated with the Master Regulator ASCL2 in Microsatellite Stability in Colorectal Cancer
Source: J Immunol Res. 2021 Feb 10;2021:5964752. doi: 10.1155/2021/5964752 (PMC7892217; doi:10.1155/2021/5964752)

Supplementary figure 1

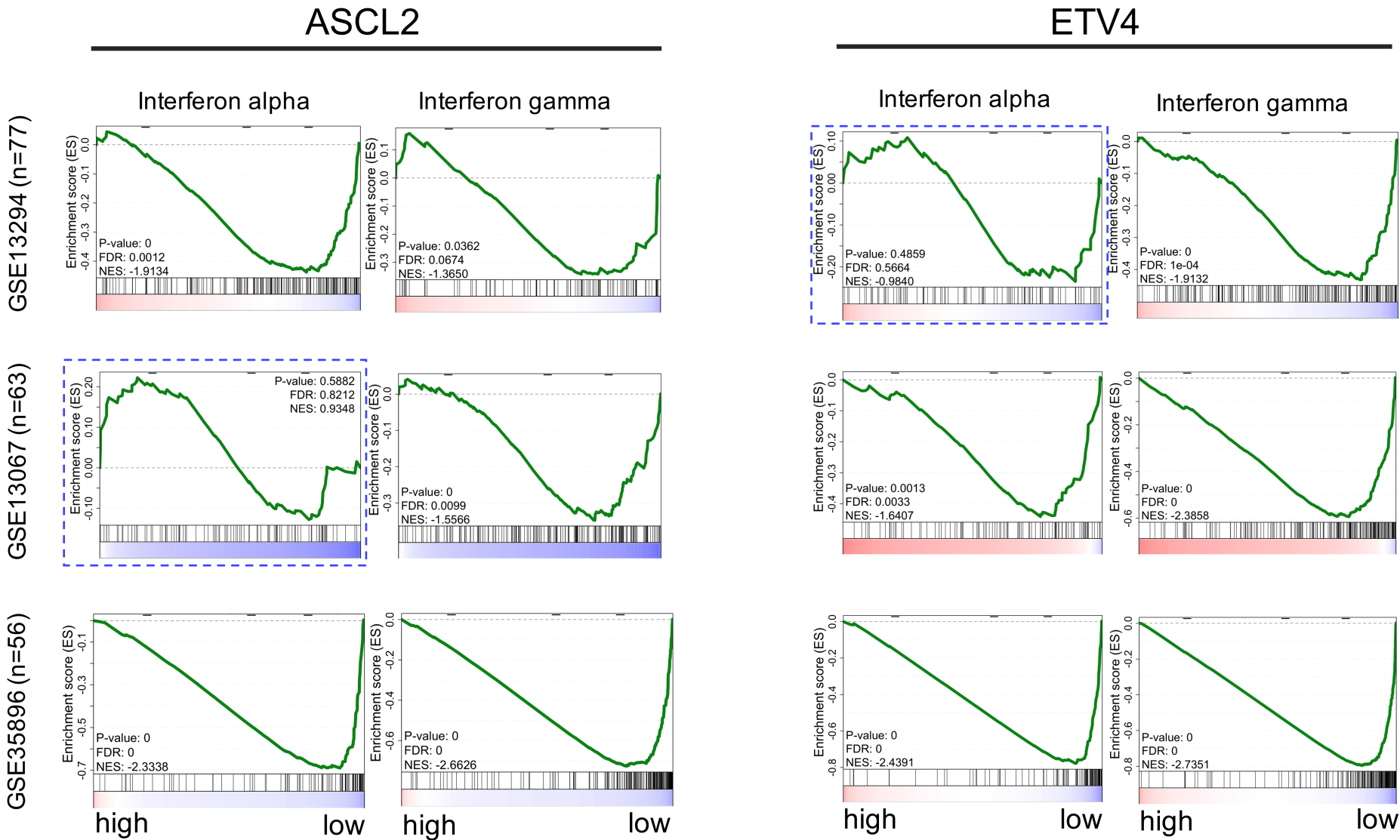

Supplement: Supplementary Materials — Supplementary Figure 1: unbiased GSEA results of ASCL2 and ETV4 based on three independent datasets of MSS CRC. Supplementary Figure 2: IGV of ETV4 locus in common GI cancers. Supplementary Table 1: leading edge genes in IFN-γ and IFN-α response pathways based on ASCL2 and ETV4 classification. Supplementary Table 2: abbreviation list ranked by the present order. [file 5964752.f1.zip › Supplementary figure 1.pdf]

Supplementary figure 2

# IGV tracks of common GI cancer H3K27Ac ChIP-Seq data

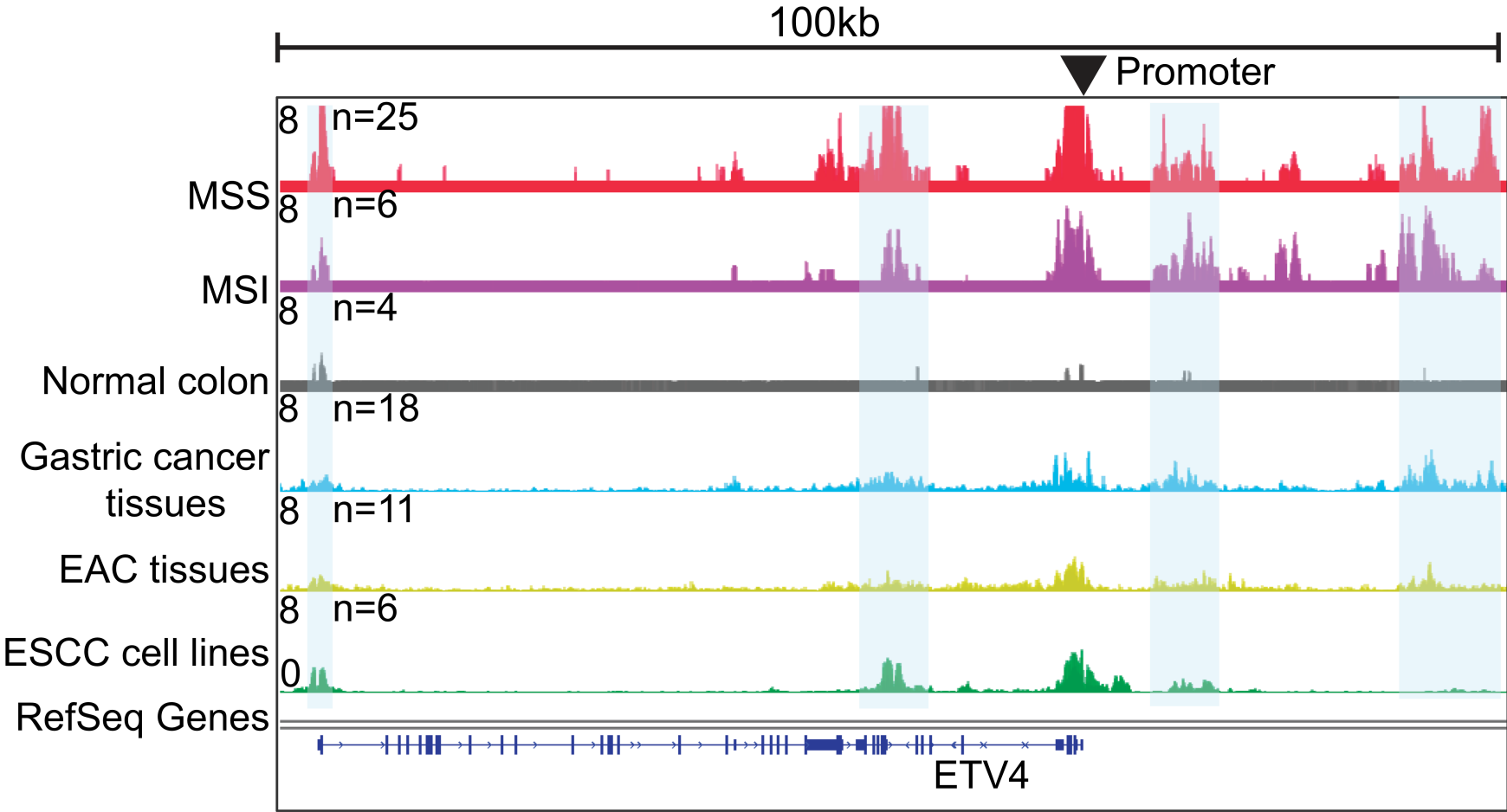

Supplement: Supplementary Materials — Supplementary Figure 1: unbiased GSEA results of ASCL2 and ETV4 based on three independent datasets of MSS CRC. Supplementary Figure 2: IGV of ETV4 locus in common GI cancers. Supplementary Table 1: leading edge genes in IFN-γ and IFN-α response pathways based on ASCL2 and ETV4 classification. Supplementary Table 2: abbreviation list ranked by the present order. [file 5964752.f1.zip › Supplementary figure 2.pdf]
